# Metagenomics Reveals Planktonic Bacterial Community Shifts across a Natural CO_2_ Gradient in the Mediterranean Sea

**DOI:** 10.1128/genomeA.01543-14

**Published:** 2015-02-12

**Authors:** Ashvini Chauhan, Ashish Pathak, Riccardo Rodolfo-Metalpa, Marco Milazzo, Stefan J. Green, Jason M. Hall-Spencer

**Affiliations:** a School of the Environment, Florida A&M University, Tallahassee, Florida, USA; b Marine Biology and Ecology Research Centre, Plymouth University, Plymouth, United Kingdom; c Institut de recherche pour le développement, Nouméa, New Caledonia; d Dipartimento di Scienze della Terra e del Mare, University of Palermo, Palermo, Italy; e DNA Services Facility, University of Illinois at Chicago, Chicago, Illinois, USA

## Abstract

Bacterial communities at a CO_2_ vent (pH 6.7) were compared with those at control (pH 8.0) and transition sites (pH 7.6) using 16S rRNA metagenomics. *Firmicutes* and unclassified bacteria dominated across all sites, *Proteobacteria*, especially *Gammaproteobacteria*, declined, and *Epsilonproteobacteria* increased in the vent with an increase in *Bacteroidetes* at both the vent and transition sites.

## GENOME ANNOUNCEMENT

The current average oceanic surface pH of 8.1 is falling rapidly, at an unprecedented rate, due to increasing anthropogenic CO_2_ emissions (1), impacting biodiversity and fundamentally altering food webs (2). It is difficult to simulate ocean acidification for sufficient periods to monitor ecosystem level effects, so submarine CO_2_ vent ecosystems are being used as “natural laboratories” to gauge acidification impacts (3–7). Because marine microorganisms recycle organic matter, making it available to higher life (8), pH shifts can have dramatic and long-lasting impacts on microbially mediated processes. We used amplicon-based metagenomic sequencing to assess planktonic microbial communities at a vent off Vulcano Island (38°25′08.52″N, 14°57′39.13″E), Italy, where gaseous emissions comprise >98% CO_2_, causing a pH gradient reaching ambient conditions (pH ~8.1), ca. 350 m from the intense CO_2_ leakage site (4).

Replicate surface water samples (*n* = 3 to 5) from the Vulcano vent ecosystem were collected from 300-m transects using a peristaltic pump into sterilized and dissolved organic carbon (DOC) clean containers. CO_2_ concentration gradients from where the samples were collected ranged from modern levels to projected late-21st-century conditions (pH ~7.6); thus, reference samples had mean pH 8.0, transition pH 7.6, and vent pH 6.7, respectively. Samples were filtered through 0.22-µm pore-size filters and shipped overnight to the Chauhan laboratory, where metagenomic DNA was extracted from the filters with a PowerWater DNA isolation kit (Mo Bio Laboratories, Inc., Carlsbad, CA, USA), and pyrotag sequencing of the 16S rRNA gene V1-V3 region was performed using standard protocols (9). Sequences obtained using a Roche 454 FLX instrument (Roche, Indianapolis, IN, USA) were processed using MOTHUR (10) as demonstrated (11), and a total of 95.1 Mb data containing 79,078 16S rRNA gene sequences were obtained and identified using RDP (12) and MG-RAST (13).

Heatmap and UniFrac analysis revealed that the vent microbiota were taxonomically distinct from those in the transition and reference samples. Interestingly, 8% to 37% of the retrieved sequences remained taxonomically unresolved, especially from the vent, indicating the presence of potentially novel bacteria within this Mediterranean ecosystem. Relative abundances of *Firmicutes* were highest across all sites (37% to 70%), which is rather unusual for marine waters. However, previous studies have, in fact, identified bacteria, such as novel Bacillus vulcani and Bacillus aeolius (14, 15), from the Gram-positive *Firmicutes* phylum from this ecosystem.

Of particular interest was the decline of *Proteobacteria*, especially *Gammaproteobacteria*, and the increase in *Epsilonproteobacteria* at the vent relative to the transition and reference sites. It is likely that the high CO_2_-low pH environment favors chemosynthetic *Epsilonproteobacteria*, many of which are human and animal pathogens (16, 17). The abundances of *Bacteroidetes* in both the vent and transition sites also increased compared with the reference site, suggesting enhanced availability of high-molecular-weight dissolved organic matter (DOM) on which *Bacteroidetes* are known to thrive (18, 19). Because the gas composition at the Vulcano seep consists of >98% carbon dioxide (4), elevated CO_2_ potentially stimulates DOM production and consumption, as demonstrated previously in mesocosms held at different CO_2_ levels (20). Further metagenomic analysis of this high CO_2_-low pH “natural laboratory” may strengthen our ability to predict the impacts of ocean acidification on marine microorganisms.

### Nucleotide sequence accession number.

The DNA sequences from this metagenomic project were deposited in the Sequence Read Archive under the accession no. SRP050984.
